# The Role of FoxG1 in the Inner Ear

**DOI:** 10.3389/fcell.2020.614954

**Published:** 2020-12-03

**Authors:** Yanyan Ding, Wei Meng, Weijia Kong, Zuhong He, Renjie Chai

**Affiliations:** ^1^Department of Otorhinolaryngology, Union Hospital, Tongji Medical College, Huazhong University of Science and Technology, Wuhan, China; ^2^Department of Otolaryngology Head and Neck, Nanjing Tongren Hospital, School of Medicine, Southeast University, Nanjing, China; ^3^State Key Laboratory of Bioelectronics, School of Life Sciences and Technology, Jiangsu Province High-Tech Key Laboratory for Bio-Medical Research, Southeast University, Nanjing, China; ^4^Co-Innovation Center of Neuroregeneration, Nantong University, Nantong, China; ^5^Institute of Stem Cell and Regeneration, Chinese Academy of Sciences, Beijing, China; ^6^Beijing Key Laboratory of Neural Regeneration and Repair, Capital Medical University, Beijing, China

**Keywords:** FoxG1, hearing loss, inner ear hair cells, autophagy, development

## Abstract

Sensorineural deafness is mainly caused by damage to the tissues of the inner ear, and hearing impairment has become an increasingly serious global health problem. When the inner ear is abnormally developed or is damaged by inflammation, ototoxic drugs, or blood supply disorders, auditory signal transmission is inhibited resulting in hearing loss. Forkhead box G1 (FoxG1) is an important nuclear transcriptional regulator, which is related to the differentiation, proliferation, development, and survival of cells in the brain, telencephalon, inner ear, and other tissues. Previous studies have shown that when FoxG1 is abnormally expressed, the development and function of inner ear hair cells is impaired. This review discusses the role and regulatory mechanism of FoxG1 in inner ear tissue from various aspects – such as the effect on inner ear development, the maintenance of inner ear structure and function, and its role in the inner ear when subjected to various stimulations or injuries – in order to explain the potential significance of FoxG1 as a new target for the treatment of hearing loss.

## Introduction

The number of people suffering from hearing impairment in the world was approximately 500 million in 2015, ranking fourth among all disability factors and ahead of diabetes and dementia (GBD, 2016). Factors that cause hearing disability include congenital, infectious, noise exposure, drugs/medications, age-related, traumatic, and immune-mediated causes. Most of these factors induce damage to the inner ear tissue and eventually cause sensorineural deafness (Brown et al., 2018). The nuclear transcription factor FoxG1 has been shown to affect the process of cell proliferation and differentiation (Ariani et al., 2008; Florian et al., 2012), and in the study of Rett syndrome it was found that FoxG1 might indirectly affect oxidative damage to erythrocytes (Ciccoli et al., 2015; Valacchi et al., 2017). In addition, researchers have found that FoxG1 has an important regulatory effect on mitochondrial energy metabolism and biosynthesis in neuroepithelial cells (Pancrazi et al., 2015). In the inner ear, FoxG1 is mainly involved in regulating the formation and differentiation of hair cells (HCs), supporting cells, and cochlear spiral neurons, thereby maintaining cochlear function and morphology (Jahan et al., 2018; He et al., 2019; Zhang et al., 2020). In addition, hearing loss caused by aging, noise exposure, and ototoxic drugs is mainly due to damage to inner ear cells caused by oxidative stress (Wang et al., 2002; Kujawa and Liberman, 2009; Liberman et al., 2015; Francis and Cunningham, 2017; Jang et al., 2018), and FoxG1 can affect the reactive oxygen species (ROS) level in cells by maintaining mitochondrial function. Therefore, exploration of the role of FoxG1 in the inner ear will increase the use of FoxG1 as a target in the treatment of hearing loss.

## The Role of FoxG1 in the Neural Stem Cells

Neural stem cells (NSCs) are self-renewable multipotent cells that can differentiate into different types of nerve cells (Chandwani et al., 2019). Because neural progenitor cells (NPCs) have limited life span and poor self-renewal ability, NSCs regulate the balance of pro-death and pro-survival signals to ensure the number of progenitor cell pools during development (Yadirgi et al., 2011; Sierra et al., 2015). As one of the markers of NPCs in the brain, FoxG1 was used to detect the differentiation level of pluripotent stem cells and embryonic stem cells (Yahata et al., 2011; Yamamizu et al., 2013). During embryonic development, FoxG1 is mainly expressed in the progenitor cells of the cerebral cortex, basal ganglia, and olfactory bulb (Dou et al., 1999). With the extension of development time, the expression area of FoxG1 changes. At mouse E12.5, FoxG1 is still expressed in the NPCs of the telencephalon, but no longer expressed in other neural tubes (Hettige and Ernst, 2019). In the mature mouse brain, FoxG1 is only expressed in neuroepithelial cells such as the cerebral cortex and hippocampus (Hettige and Ernst, 2019). These indicate that FoxG1 can regulate the telencephalic development through spatio-temporal patterning and interaction with different signaling. In the telencephalon of *Foxg1* null mice, the dorsal neuroepithelial cells proliferation reduced and differentiate prematurely, and lead to depletion of the NPC pool (Xuan et al., 1995; Hanashima et al., 2002; Martynoga et al., 2005). This indicate that FoxG1 is involved in regulating the neuroepithelial cell proliferation and differentiation time during the morphogenesis and development of the telencephalon. Researchers used reprogramming approach to transduces FoxG1 and other transcription factors into mouse fibroblasts and astrocytes and they successfully converted somatic cells into proliferative NPCs (Ma et al., 2019). Brancaccio et al. (2010) found that overexpression of *Foxg1* gene can maintain the ability of cells self-renewal and promote the increase in the number of NSCs. The self-renewal activity of NSCs decreases with age. Nakatani et al. (2019) found that the expression of *Ecrg4* gene was significantly increased in aging NSC. When the *Ecrg4* was overexpressed in NSC, the proliferation ability of NSC was significantly reduced, and when the expression of Ecrg4 was deleted, the decline in the proliferation ability of NSC caused by age was recovered. The NSC proliferation caused by the deletion of *Ecrg4* expression was achieved by activating the expression of *Foxg1* (Nakatani et al., 2019).

In the inner ear, after conditional knockout *Foxg1* in the HCs, we found that the number of HCs in the apex turn of the cochlea of newborn mice increased significantly, indicating that the deletion of *Foxg1* expression caused the disorder of HC proliferation and differentiation (He et al., 2019). By transcriptome sequencing analysis of HCs, we found that the knockout of *Foxg1* caused abnormal expression of multiple signaling pathways and related genes. The knockout of *Foxg1* caused the inhibition of the Notch signaling pathway in HCs, which probably led to the premature differentiation of NPCs, and ultimately resulted in the increase of HCs in the apex turn of the cochlea of newborn mice (He et al., 2019). After conditional knockout *Foxg1* in supporting cells and inner ear stem cells using Sox2-CreER mice and Lgr5-EGFP-CreERT2 mice, we also found that the number of HCs increased significantly (Zhang et al., 2020). Through EDU assay and *in vitro* sphere-forming assay, we found that this phenotype was mainly due to the knockout of *Foxg1* promoting the *trans-*differentiation of supporting cells to HCs, and the expression of genes related to the cell cycle and Notch signaling pathway was also affected (Zhang et al., 2020). The above findings indicated that FoxG1 can affect the differentiation and proliferation of inner ear NPCs through the regulation of multiple signal pathways and related factors expression.

## The Role of FoxG1 in Other Tissues

**FIGURE 1:**
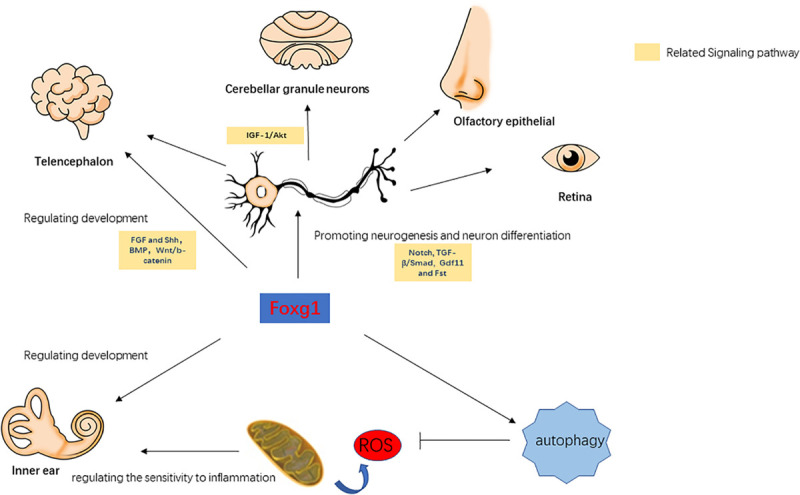
The role of FoxG1 in the development, differentiation, and survival of various tissues.

Forkhead box G1 belongs to the Fox transcription factor gene family, and is involved in the regulation of telencephalon development, cortical neuron differentiation, neurogenesis, and axonal exogenous growth (Brancaccio et al., 2010; Manuel et al., 2011; Figure 1). In *Foxg1* knockout mice, it was found that when the expression of FoxG1 in the embryonic stage was suppressed the volume of the cerebral hemisphere was severely reduced and the mice died soon after birth, and large numbers of precursor cells differentiated into Cajal-Retzius cells and the differentiation into cortical neurons was inhibited, resulting in thinning of the cortex and abnormal neuronal stratification (Hanashima et al., 2004, 2007; Cargnin et al., 2018; Testa et al., 2019). In the development of the ventral telencephalon, the absence of FoxG1 expression causes abnormal expression of FGF and Shh signaling-related pathways, which affect the development of the ventral telencephalon (Manuel et al., 2010). This indicates that FoxG1 may affect the development of the telencephalon by regulating other signal pathways. In the telencephalon, dentate gyrus, and cerebral cortex, FoxG1 has been found to play an important role in regulating the proliferation and differentiation of neural progenitor cells. When FoxG1 expression is absent, this leads to cell cycle disruption in the progenitor cells of the telencephalon (Manuel et al., 2011), a decrease in the population of cortical intermediate progenitor cells (Siegenthaler et al., 2008), and a decrease in the number of stem cells due to the loss of the self-renewal capacity of neural stem cells (Brancaccio et al., 2010). In addition, FoxG1 is also involved in regulating the integration of multipolar pyramidal neuronal precursors into cortical plates (Miyoshi and Fishell, 2012). In the development of the olfactory epithelium, FoxG1 can affect the proliferation and differentiation of olfactory epithelial cells in cooperation with the Gdf11 (Growth differentiation factor 11) and Fst (Follistatin) proteins (Kawauchi et al., 2009). In the development of the chicken brain, the overexpression of FoxG1 can lead to the massive outgrowths of telencephalon and mesencephalon. This phenotype was not due to the activation of cell proliferation after the increase in FoxG1 expression, but due to the inhibition of apoptosis (Ahlgren et al., 2003). When FoxG1 expression is abnormal, nerve development in tissues such as the cerebral cortex, telencephalon, ear, retina, and olfactory epithelium is inhibited (Pauley et al., 2006). Since the inner ear and the above tissues have the same neurodevelopmental process, and FoxG1 has an important regulatory role in other tissues, we believe that it may also have a similar important regulatory role in the inner ear.

## FoxG1-Related Signaling Pathways

Forkhead box G1 plays a cooperative regulatory role with the IGF-1/Akt, TGF-β/Smad, BMP, Wnt/β-catenin, Notch, and other signaling pathways. Dastidar et al. (2012) found that normal expression levels of FoxG1 can inhibit apoptosis in mouse cerebellar granule neurons, and when FoxG1 is overexpressed it has an antagonistic effect on the pro-survival factor IGF-1. TGF-β can inhibit the proliferation of a variety of embryonic epithelial cells, and FoxG1 was found to have an antagonistic effect on TFG-β (Pelton et al., 1991; Feijen et al., 1994; Furuta et al., 1997). When FoxG1 expression is inhibited, TGF-β or other growth inhibitory factors enhance the inhibitory effect on brain precursor cells (Pelton et al., 1991). In the TGF-β/Smad pathway, the binding of Smad to its ligands is competitively inhibited by FoxG1, thereby inhibiting the function of Smad. When FoxG1 competitively binds with Smad, this also suppresses the expression of TGF-β target genes, thereby blocking its downstream pathway (Dou et al., 2000). BMP can enhance the differentiation efficiency of precursors in the telencephalon, while FoxG1 can affect cellular differentiation by inhibiting the expression of BMP (Liu et al., 2018). Wnt/β-catenin and Shh can regulate the differentiation of the ventral and dorsal telencephalon, and Danesin et al. (2009) found that FoxG1 can directly or indirectly inhibit the expression of the Wnt/β-catenin pathway, thereby affecting cellular differentiation in the telencephalon. In addition, FoxG1 also has a regulatory relationship with Hes, Groucho/TLE, and other Notch signaling pathways, and FoxG1 can prevent the premature differentiation of precursor cells by inhibiting the expression of genes related to neurogenesis targeted by the Notch signaling pathway (Dali et al., 2018; Chiola et al., 2019; Richard and Jia-Hao, 2020; Zhang et al., 2020). In summary, FoxG1 affects multiple physiological processes and is associated with multiple signaling pathways. FoxG1 maintains the normal growth and development of various tissues by regulating cell proliferation and apoptosis (Figure 1). In the survival and development of inner ear HCs, the above-mentioned signaling pathways also play an important regulatory role. For example, the Wnt and Notch signaling pathway are related to the development of the inner ear, the generation and differentiation of supporting cells, HCs and neurons (Jayasena et al., 2008; Jacques et al., 2012; Brown et al., 2020). BMP signaling pathway affects inner ear morphogenesis, nerve fiber formation, and HC development (Yang et al., 1999; Blauwkamp et al., 2007). Therefore, FoxG1 is likely to affect the development of the inner ear and the survival of HCs through interaction with these pathways.

## The Role of FoxG1 in the Inner Ear

### The Role of FoxG1 in Inner Ear Development

**FIGURE 2:**
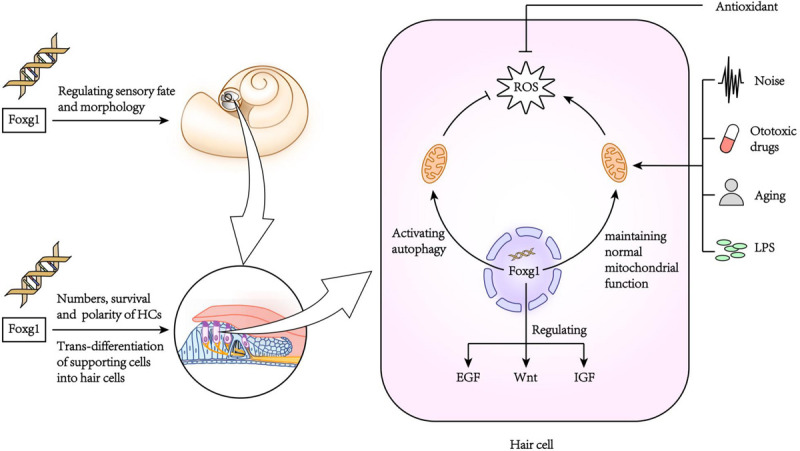
The role of FoxG1 in the inner ear.

Pauley et al. (2006) found that FoxG1 was expressed in most types of cells in the crista, endolymphatic vessels and organ of Corti of the inner ear, including HCs, supporting cells, border cells, and Hensen cells, etc. FoxG1-deletion mice had significantly shorter cochlear ducts than normally developing mice and lacked the formation of the horizontal crista and ampulla (Pauley et al., 2006). In addition, the distribution of inner ear nerve fibers in FoxG1-deletion mice also showed abnormalities (Harasztosi et al., 2019). Hwang et al. found that FoxG1 is essential for the formation and separation of the sensory cristae, indicating that FoxG1 has a function in regulating sensory fate in the inner ear (Hwang et al., 2009). Deletion of FoxG1 can also cause the polarity of HCs to change (Pauley et al., 2006). Our previous research found that after specifically knocking out FoxG1 in HCs, the cochlear ducts length did not change after birth, but there was an increase in the number of HCs (He et al., 2019). In addition, we also found that the Wnt, IGF, and EGF signaling pathways were inhibited in HCs absent FoxG1 expression, and the survival time of adult mouse HCs was shortened (Figure 2; He et al., 2019).

### The Role of FoxG1 in Cell Survival in the Inner Ear

Due to the non-renewability of mammalian cochlear HCs, their damage is the main cause of hearing loss. When FoxG1 expression is inhibited, it will not only cause morphogenesis and functional defects of the auditory system, but also affect the survival of HCs and ultimately lead to hearing loss. In newborn mouse cochleae with conditional knockout *Foxg1* in HCs, we found that the HCs in the apex turn of the mouse cochlea increased significantly at P1–P7, and then gradually returned to normal levels with the increase of time, and there was no significant change in the hearing of the mice before P21 (He et al., 2019). But after P21, the HCs in the basal turn appeared to be lost, and as the mouse ages, the number of HCs lost gradually increases and develops toward the apex turn. In addition, the results of RNA sequencing analysis showed that the deletion of *Foxg1* expression cause the inhibition of IGF signaling pathway in HCs (Figure 2). Previous studies have found that FoxG1 and IGF1 have a synergistic regulatory effect. IGF1 plays an important role in the protection of nerve cell damage, that is, the inner ear cells of IGF1 knockout mice will appear apoptosis and hearing loss (Camarero et al., 2002; Murillo-Cuesta et al., 2011). Therefore, FoxG1 may affect cell proliferation and apoptosis sensitivity by regulating multiple signal pathways such as IGF and Notch. de Iriarte Rodríguez et al. (2015) found that abnormal expression of C-Raf caused hearing loss and increased sensitivity to noise in mice. In the embryonic cochlea of *C-Raf* null mice, the expression of *Foxg1* increased. It is speculated that this phenomenon is due to the body promotes the survival of auditory neurons in the inner ear through activating the expression of *Foxg1*, thereby reducing the cochlea development abnormal caused by the deletion of *C-Raf* expression.

### The Role of FoxG1 in Inflammation in the Inner Ear

The inner ear is often stimulated by various factors leading to an inflammatory reaction. A mild inflammatory reaction can remove toxins and pathogenic microorganisms, and thus has a protective effect on tissues and cells, but excessive inflammatory reactions can cause serious damage to the inner ear (Kalinec et al., 2017). In the model of inflammation of the inner ear caused by LPS (lipopolysaccharide), inflammatory cells accumulate in the inner ear causing the stria vascularis to swell, which in turn damages the auditory HCs (Hirose et al., 2014; Hirose and Li, 2019). In our previous research, we found that FoxG1 has an important regulatory role in inhibiting the sensitivity of aging HCs to inflammation (He et al., 2020b). When the HCs were treated with low concentration of LPS, the expression level of FoxG1 and autophagy increased, on the contrary, when treated with high concentration of LPS, the levels of both decreased significantly, which indicated that FoxG1 may play its role in promoting survival through the regulation of autophagy. When the expression of FoxG1 was inhibited, the level of autophagy in HCs was also inhibited, and the level of apoptosis was significantly increased. We also found that in D-galactose-induced aging HCs, FoxG1 inhibits the increase in ROS in cells induced by LPS by activating autophagy, thereby regulating the sensitivity of aging HCs to inflammation and maintaining the function and survival of HCs (He et al., 2020a; Figure 2).

## Discussion

Our research on FoxG1 in the inner ear suggests that FoxG1 may be involved in protecting the inner ear from damage (He et al., 2020a). Ototoxic drugs and aging are the two main causes of inner ear damage and mainly include aminoglycoside antibiotics (such as neomycin and gentamicin) and anti-tumor platinum-based drugs (such as cisplatin) (Ryals et al., 2018). For mechanism of ototoxic drugs inducing deafness, Schacht, 1999 found that ototoxic drugs mainly damage the inner ear cells by generating excessive oxygen free radicals, which would injury hearing function finally, and the use of antioxidants can reduce such damage. In addition, related studies on noise-induced hearing loss have found that oxidative stress caused by noise is also an important cause of cellular damage in the inner ear (Ohlemiller, 2008), and Shuhei and Xiangxin found that IGF-1 can effectively inhibit neomycin-induced damage to HCs (Yoshida et al., 2015; Xiangxin Lou et al., 2015 Jan). It is known that FoxG1 not only inhibits the increase in ROS level by activating autophagy, but also has a close regulatory relationship with IGF-1. Therefore, FoxG1 might be involved in regulating the processes through which ototoxic drugs and noise exposure damage the inner ear.

It is now generally accepted that age-related oxidative stress is one of the factors leading to hearing loss (da Costa et al., 2016). When mitochondrial function is abnormal, the excessive ROS in the cell will disrupt gene expression, protein renewal, and other biological functions, which will lead to disrupted biosynthesis and energy metabolism and eventually lead to cell death. It is known that FoxG1 affects mitochondrial membrane potential and mitochondrial division and fusion (Pancrazi et al., 2015), and when FoxG1 expression is abnormal mitochondrial energy metabolism, biosynthesis, and membrane potential are disturbed, which in turn affects cell proliferation and differentiation. Therefore, normal expression of FoxG1 is a key factor in maintaining normal mitochondrial function and ROS levels. Rodriguez-de la Rosa et al. found that IGF-1 not only has anti-apoptotic effects, but also can activate cell renewal (Rodriguez-de la Rosa et al., 2017). Mariño et al. found that IGF-1 can extend the lifespan of premature aging mice (Zmpste24-deficient mice) by restoring somatotroph axis function (Mariño et al., 2010). Therefore, FoxG1 might affect the occurrence and development of age-related hearing loss by regulating multiple pathways. *Foxg1* as one of the marker genes of inner ear progenitor cells and plays an important role in the process of inducing pluripotent stem cells to differentiate into inner ear cells (Boddy et al., 2020). Therefore, the *Foxg1*-related reprogramming technology has great application value to regenerate cells in the inner ear that are affected by pathology or damage. C-MYC is a regulatory factor that plays an important role in cell proliferation, growth and apoptosis (Han et al., 2009). In the study of wound repair, Zhan et al. (2018) found that nitric oxide can induce the transcription of c-myc to promote the proliferation of epidermal stem cells, and the c-myc promoter activity is regulated by FoxG1 during this process. In the related research of the inner ear, it was found that c-myc can not only protect the inner ear from noise damage but also promote the self-renewal of otic progenitor cells (Han et al., 2009; Kwan et al., 2015). The loss of sensory HCs and neurons in the inner ear is the main cause of sensorineural hearing loss, and this loss is irreversible. The promoters of c-myc and Sox2 are highly similar, and the target genes include kinases that regulate the cell cycle (Kwan et al., 2015). Therefore, FoxG1 may play an important role in the regeneration of HCs by regulating the c-myc signaling pathway. However, there are relatively few studies on FoxG1 in the inner ear. Moreover, FoxG1 may have different regulatory mechanisms in different organs and tissues, as well as in the growth and development of different types of cells. Therefore, the regulation mechanism of FoxG1 in the inner ear remains to be studied in the future.

## Conclusion

Forkhead box G1 not only plays a key role in the development of the cerebral cortex and neurons, but also has a close regulatory relationship with the development of the inner ear, the survival of HCs, and the protection of HCs against injury. FoxG1 is essential for the maintenance of NSCs and NPCs, and directly regulates the differentiation process of cells. It has been reported that FoxG1 can promote the survival of inner ear HCs by regulating autophagy, mitochondrial function, and related signaling pathways. Thus the in-depth exploration of the role of FoxG1 in the inner ear will improve its use as a target for the regeneration of HCs and the treatment of sensorineural hearing loss.

## Author Contributions

YD, WM, and ZH conceived and wrote the manuscript. WK, ZH, and RC modified the manuscript. All authors contributed to the article and approved the submitted version.

## Conflict of Interest

The authors declare that the research was conducted in the absence of any commercial or financial relationships that could be construed as a potential conflict of interest.

## Abbreviations

FoxG1: Forkhead box G1
ROS: reactive oxygen species
IGF-1: insulin like growth factor-1
TGF-β: transforming growth factor beta
BMP: bone morphogenetic protein
HCs: hair cells
LPS: lipopolysaccharide
NPCs: neural progenitor cells
NSCs: neural stem cells.
